# Endoplasmic reticulum stress activation during total knee arthroplasty

**DOI:** 10.1002/phy2.52

**Published:** 2013-08-22

**Authors:** Austin D Hocker, Ryan M Boileau, Brick A Lantz, Brian A Jewett, Jeffrey S Gilbert, Hans C Dreyer

**Affiliations:** 1 Department of Human Physiology, University of OregonEugene, Oregon; 2 Slocum Center for Orthopedics and Sports MedicineEugene, Oregon

**Keywords:** Clinical, ER stress, ischemia reperfusion, muscle, unfolded protein response

## Abstract

Total knee arthroplasty (TKA) is the most common remediation for knee pain from osteoarthritis (OA) and is performed 650,000 annually in the U.S. A tourniquet is commonly used during TKA which causes ischemia and reperfusion (I/R) to the lower limb but the effects of I/R on muscle are not fully understood. Previous reports suggest upregulation of cell stress and catabolism and downregulation of markers of cap-dependent translation during and after TKA. I/R has also been shown to cause endoplasmic reticulum (ER) stress and induce the unfolded protein response (UPR). We hypothesized that the UPR would be activated in response to ER stress during TKA. We obtained muscle biopsies from the vastus lateralis at baseline, before TKA; at maximal ischemia, prior to tourniquet deflation; and during reperfusion in the operating room. Phosphorylation of 4E-BP1 and AKT decreased during ischemia (−28%, *P* < 0.05; −20%, *P* < 0.05, respectively) along with an increase in eIF2α phosphorylation (64%, *P* < 0.05) suggesting decreased translation initiation. Cleaved ATF6 protein increased in ischemia (39%, *P* = 0.056) but returned to baseline during reperfusion. CASP3 activation increased during reperfusion compared to baseline (23%, *P* < 0.05). *XBP1* splicing assays revealed an increase in spliced transcript during ischemia (31%, *P* < 0.05) which diminished during reperfusion. These results suggest that in response to I/R during TKA all three branches of the ER stress response are activated.

## Introduction

Total knee arthroplasty (TKA) is used to mitigate knee pain caused by osteoarthritis that affects 60% of the U.S., over the age of 65 (Parsley et al. 2010) and is the leading cause of hospitalization for adults ages 45–84 years (Pfuntner and Stocks 2013). In 2008 over 650,000 TKAs were performed in the U.S. at a cost of $9 billion (Kurtz et al. 2011; Cram et al. 2012). The prevalence of TKA is projected to increase to 3.5 million annually if growth rates remain constant as they have in the last 15 years (Kurtz et al. 2011). Despite success as a treatment of osteoarthritis TKA leaves patients with persistent muscle atrophy and loss of function. Muscle atrophy of the knee extensors is responsible for the majority of functional deficit 1–3 years post-TKA (Meier et al. 2009) by inhibiting balance (Moxley Scarborough et al. 1999), reducing functional mobility (Brown et al. 1995; Mizner et al. 2005c), and increasing the risk of falls (Moreland et al. 2004).

Acute stoppage of blood flow occurs with tourniquet use during TKA to create a bloodless field. Before tourniquet application, the leg is elevated and an Esmarch bandage is applied in a distal to proximal fashion to expel blood from the operative limb. The tourniquet is inflated above arterial pressure stopping flow and causing ischemia to the lower limb. The effects of ischemia followed by reperfusion (I/R) on human skeletal muscle metabolism is poorly understood at the cellular level.

Although several early studies suggested there was little effect of I/R on skeletal muscle, other studies have shown muscle may be susceptible to ischemic periods of 15–60 min (Suval et al. 1987a,b; Sexton et al. 1990; Sternbergh and Adelman 1992; Duarte et al. 1997; Racz et al. 1997; Appell et al. 1993). Work from our lab has shown muscle cell signaling alterations during I/R suggesting upregulation of the catabolic FOXO3A and SAPK/JNK cell stress pathways (Bailey et al. 2012) as well as downregulation of cap-dependent translation and anabolic pathways (Ratchford et al. 2012). This imbalance in protein anabolism and catabolism suggest a potential mechanism contributing to early stages of the rapid muscle atrophy following TKA.

Ischemia-reperfusion has been shown to induce endoplasmic reticulum (ER) stress in a variety of tissues including smooth and cardiac muscle (Treiman 2002) but not skeletal muscle. ER stress is caused by accumulation of unfolded proteins in the ER due to disruption of protein folding capacity, usually induced by calcium dysregulation, decreased protein glycosylation, or lipid biosynthesis dysregulation. Ischemia alters calcium kinetics and glycolysis altering the ability of proteins to fold in the ER (Gorlach et al. 2006). Unfolded proteins are sensed in the ER lumen by the chaperone protein binding Ig protein (BIP)/GRP78. BIP has a high affinity for unfolded hydrophobic stretches of polypeptides and when unfolded proteins accumulate BIP is drawn away from the ER membrane where it sequesters PKR-like endoplasmic reticulum kinase (PERK), inositol requiring element 1α (IRE1α), and activating transcription factor 6 (ATF6). PERK leaves the ER membrane, oligomerizes and phosphorylates eIF2α at Ser51 inhibiting translation initiation and selectively enhancing ATF4 transcription. ATF6 is shuttled to the golgi where it is cleaved and activated leading to activation of ERAD (Endoplasmic reticulum-associated protein degradation) genes. After it is released from BIP, IRE1α targets *XBP1* mRNA via a ribonuclease domain which splices out a 26nt hairpin from the *XBP1* transcript changing its reading frame. This change in reading frame translates to a spliced XBP1 (XBP1s) protein with a C-terminal end that is an active bZIP transcription factor and a stimulator of UPR target genes (Yoshida et al. 2001). Together these three branches of the unfolded protein response (UPR) act to alleviate ER stress by downregulating protein synthesis and upregulating chaperones and folding proteins in the ER (Deldicque et al. 2012).

Endoplasmic reticulum stress is also implicated in the development of apoptosis through IRE1α signaling. IRE1α activates ER membrane associated initiator caspases that activate terminal, apoptotic caspases (Nakagawa et al. 2000; Morishima et al. 2002) such as caspase 3 (CASP3) that has been shown to play an important role in initializing muscle protein degradation in catabolic conditions by cleaving actin and myosin proteins for degradation by the ubiquitin-proteosome system (Du et al. 2004). Interestingly, ER stress induced apoptosis is protective acutely (Ogata et al. 2006) but may induce apoptosis during severe stress (Lin et al. 2007; Gardner et al. 2013). Furthermore, recent studies show dephosphorylation of 4E-BP1 and AKT during ER stress corresponding to anabolic resistance and decreased protein synthesis. Together, these studies implicate ER stress in the maintenance of muscle mass through modulation of protein synthesis and breakdown which may effect maintenance of muscle mass acutely after TKA.

As such, the objective of this study was to measure components of the three branches of the UPR during tourniquet-induced ischemia and subsequent reperfusion in a population undergoing TKA. We tested the hypothesis that ischemia and reperfusion during TKA would activate the UPR in response to ER stress.

## Methods

### Ethics approval

This study was approved by the PeaceHealth Institutional Review Board, Sacred Heart Medical Center, at RiverBend and the Biomedical Institutional Review Board for the University of Oregon and conducted in accordance with the Declaration of Helsinki. All subjects gave informed written consent prior to study participation. This study is registered with ClinicalTrials.gov (NCT00760383).

## Subjects

We recruited male and female subjects between 60 to 80 years of age (*n* = 13) from a pool of surgical candidates from the Slocum Center for Orthopedics and Sports Medicine. All subjects were scheduled to undergo a primary TKA, had no currently untreated endocrine disease, significant heart, kidney, liver, blood or respiratory disease, peripheral vascular disease, active cancer, recent treatment with anabolic steroids or oral corticosteroids for greater than 1 week, and no alcohol or drug abuse. Subject characteristics are provided for each subject in Table 1.

**Table 1:** Physical characteristics of subjects

Sex	Age	Ht (cm)	Wt (kg)	BMI	Dx	Medications	Tourniquet time (min)	Reperfusion time (min)	Anesthesia
F	65	160	79.8	31.2	Deg Arth	Aleve, Lexapro, Wellbutrin	41	17	Spinal, FNB
F	70	157	99.8	40.2	OA	Betimol, Caltrate, HCTZ, Levothyroxine, Monopril, Tylenol, Naprosyn, Loratidine, Meclizine	49	13	Spinal
M	72	171	123.4	42.0	OA	Calcium with Vitamin D, Combigan, Enalapril, Furosemide, Glucosamine, Norvasc, Prozac, Simvastatin, Travatan, Vicodin	45	22	Gen.
F	70	157	72.6	29.3	OA	Crestor, Cephalex, Levothyroxin, Aleve, Triamterene/HCTZ	33	20	Spinal, FNB
F	79	156	49.9	20.4	OA	TriamtereneHCTZ, Vicodin, Aspirin, Lovastatin, Omeprazole, Sertraline, Naproxen	44	15	Gen.
M	76	183	83.9	25.1	OA	Aspirin, Diclofenac, Ibuprofen	51, 50	11, 14	Gen, Epd
M	65	170	98.9	34.1	OA	Pravastatin, Advil, Clopidogrel, Diltiazem, Lisinopril HCTZ, Metformin, Novolin	48	25	Gen.
F	65	157	80.7	32.6	OA	Allopurinol, Lasix, Omeprazole, Simvastatin, Tylenol	34	19	Gen.
F	64	170	113.4	39.2	OA	Tylenol, Baclofen, HCTZ, Levothyroid, Oxycodone, Oxycontin, Vasotec, Wellbutrin, Allegra	42	14	Gen.
M	70	178	116.1	36.6	OA	Levonox, Aspirin, Norco, Losartanhydrochlorothiazide, Simvastatin, Vardenafil, Diclofenac Sodium	44	15	Gen.
M	73	157	99.8	40.2	OA	Allopurinol, Aspirin, Colchicine, Lisopril	44	16	Spinal
M	71	170	86.2	29.8	OA	Aspirin, Lisinopril, Omeprazole, Simvastatin, Naproxen	60, 47	26, 25	Gen, Epd
F	68	170	79.4	27.4	OA	Aspirin, Hydrochlorothiazide, Lipitor	36	18	Spinal

The following medications were not taken for 7 days prior to surgery: aspirin, naproxen, aleve, ibuprofen.

FNB, femoral nerve block; Gen, general anesthesia; Spinal, spinal anesthesia; Epd, epidural anesthesia.

Femoral nerve block: 30 mL of 0.25–0.5% bipivicaine or ropivacaine.

General anesthesia: intravenous propofol and maintained by inhalation of either desflurane or sevoflurane.

Spinal anesthesia: 0.75% bupivicaine + 20 μg of fentanyl.

Epidural anesthesia: 0.25% bupivicaine.

Muscle relaxant: Administered by local injection of rocuronium bromide.

### Study design

Details of the study design have been published previously (Bailey et al. 2012; Ratchford et al. 2012). One week prior to surgery all subjects refrained from taking NSAIDs and aspirin. On the morning of surgery, subjects were admitted to Sacred Heart Medical Center at Riverbend in a fasted state. Anesthesia was administered with either a epidural, spinal, or general anesthetic, intravenous propofol, and inhalational anesthetic (desflurane or sevoflurane), with or without muscle relaxant (rocuronium bromide) (Table 1). After exsanguination a 10 cm wide Zimmer tourniquet was positioned around the proximal thigh of the surgical limb. The first muscle biopsy was performed prior to tourniquet inflation from the vastus lateralis on the operative leg using a 5 mm Bergstrom biopsy needle with applied suction as previously detailed. The tourniquet was inflated to 300 mmHg or greater depending on systolic blood pressure. After completion of the primary components of the surgery a second muscle biopsy was obtained prior to tourniquet deflation at maximal ischemia. This biopsy was obtained directly from the exposed muscle. The final muscle biopsy was obtained in the operating room after 18 ± 1 min of reperfusion through the same incision as biopsy #1 with the needle penetration angle and depth altered so that the site was different at each time point. Muscle biopsies were blotted to remove blood and dissected away from adipose tissue before being frozen in liquid nitrogen and stored at −80°C until analysis. Data from five subjects have previously been published (Bailey et al. 2012).

### Whole-muscle homogenization

Details for the homogenization procedures have been previously published (Bailey et al. 2012; Ratchford et al. 2012). Frozen muscle samples (40–50 mg) were crushed using Heidolph Brinkmann Silent Crusher M in homogenization buffer containing: 50 mmol/L Tris-HCl, 250 mmol/L mannitol, 50 mmol/L Na pyrophosphate, 1 mmol/L EDTA, 1 mmol/L EGTA, 1.0% Triton X-100, pH 7.4, 1 mmol/L benzamidine, 1 mmol/L DTT, 0.1 mmol/L PMSF, and 5μP/mL soybean trypsin inhibitor (1:9 weight/volume). Samples were centrifuged at 2800 ***g*** at 4°C and the supernatant was collected. Protein concentration was determined in duplicate using a Qubit 2.0 Fluorometer (Invitrogen, Carlsbad, CA).

### SDS PAGE and Immunoblotting

Details of the immunoblotting procedures have been previously published with specific (Welinder and Ekblad 2011) modifications implemented for this study (Bailey et al. 2012; Ratchford et al. 2012). Homogenates were loaded in duplicate into TGX all kDa precast gels (Bio-Rad, Hercules, CA) in electrode buffer (0.3% Tris Base, 14.4% Glycine, 1% SDS in dd-H2O) alongside a loading control for between-blot normalization. Following SDS-PAGE, proteins were transferred to PVDF membranes using Bio-Rad Trans-Blot Turbo Transfer system using the Bio-Rad Midi format at 25V for 7 min. Blots were stained with Ponceau-S as a loading control and to confirm efficient transfer (Gilbert et al. 2007; Romero-Calvo et al. 2010; Bailey et al. 2012; Ratchford et al. 2012). Informed by prior results showing total protein levels for actin, AKT, and 4E-BP1 do not change over time under identical conditions (Bailey et al. 2012; Ratchford et al. 2012), our present analysis focused on the changes in phosphorylation status of proteins of interest. Our rationale is that due to the relatively small amount of muscle obtained during each biopsy, priority was given to fully characterizing the effects of I/R on ER stress related pathways; as opposed to expressing each phosphoprotein relative to total protein, which limits the total number of proteins per blot that we could assay and because we have previously demonstrated that total protein levels do not change (Bailey et al. 2012; Ratchford et al. 2012). As such, data are presented as phosphorylation status relative to total protein content/lane normalized between blots and as percent change from baseline.

### Antibodies

The primary antibodies p-Akt Ser473 (#9271), p-4E-BP1 Thr37/46 (#9459), BCL2 (#2872), p-eIF2α Ser51 (#3597), BIP (#3177), CASP3 (#9662), CASP7 (#9492) Cell Signaling (Beverly, MA), ATF6α (#22799) Santa Cruz Biotechnologies (Santa Cruz, CA), XBP1 (#37152) Abcam (Cambridge, MA). ECL+ Anti-Rabbit IgG, horseradish peroxidase from donkey and mouse were purchased from GE Healthcare.

### Total RNA isolation and cDNA synthesis

Skeletal muscle samples (10–20 mg) were homogenized in 700 μL Qiazol (Qiagen, Venlo, Netherlands) using Heidolph Brinkmann's Silent Crusher M at 15 k rpm in RNase-free tubes. Separation was achieved through addition of 140 μL chloroform and precipitation with 0.5 mL isopropanol. The RNA pellet was washed twice in 75% ethanol, dried, and then dissolved in 1.5 μL of 0.1 mmol/L EDTA/mg of tissue. RNA concentration was determined in duplicate using a Qubit fluorometer (Invitrogen) and cDNA was reverse transcribed from 1 μg RNA using iScript Reaction Mix (Bio-Rad) according to manufacturer's instructions and stored at −80°C for analysis.

### PCR analysis of XBP1 splicing

*XBP1* mRNA was amplified by 45 PCR cycles using primers designed around the splice site as described at http://saturn.med.nyu.edu/research/mp/ronlab/protocols/XBP-1.splicing.06.03.15.pdf (FWD: 5′AAACAGAGTAGCAGCTCAGACTGC REV: 5′TCCTTCTGGGTAGACCTCTGGGAG). Amplification yields products of 472 and 448 nucleotides for XBP1u and XBP1s, respectively, depending on IRE1α splicing activity causing removal of a 26 nucleotide hairpin from XBP1u. PCR products were resolved on a 2.5% agarose gel with ethidium bromide staining and quantified densitometrically.

### Statistical analysis

Statistical evaluation of data was performed using a repeated-measures ANOVA with Mauchly's test of sphericity and Greenhouse-Geisser correction to compare ischemic and reperfusion samples percent change from baseline. Differences between means were considered significant at *P* ≤ 0.05. Differences between means were considered a trend at *P* ≤ 0.10. Analysis for all variables were performed using SPSS (v.20, IBM, Armonk, NY). All values are expressed as mean percent change ± SE, unless otherwise stated.

## Results

Subject characteristics are presented in Table 1. The average age for our cohort was 69.8 ± 1.2 years. The average BMI was 32.9 ± 1.8. The lengths of ischemia and reperfusion time were 45 ± 2 and 18 ± 1 min, respectively.

**Figure 1:**
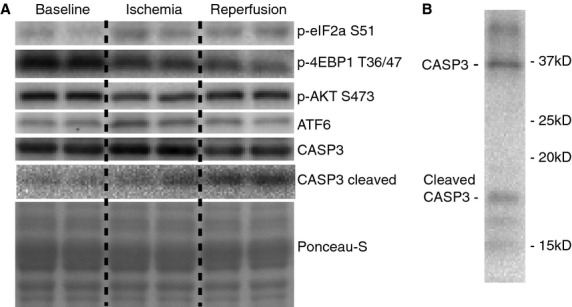
Representative protein blots. (A) Representative Western blot images for phosphorylated eukaryotic initiation factor 2α at Ser51 (p-eIF2α S51), phosphorylated eIF4E-binding protein 1 at Thr36/47 (p-4EBP1 T36/47), phosphorylated protein kinase B at Ser473 (p-AKT S473), activating transcription factor 6 (ATF6), caspase-3 (CASP3), cleaved CASP3, and loading control (Ponceau S) for baseline (immediately prior to total knee arthroplasty start); ischemia (prior to tourniquet deflation); and reperfusion (before final closure of surgical site in the operating room). (B) Representative Western blot images for CASP3 cleavage showing cleaved and full-length CASP3 bands.

**Figure 2:**
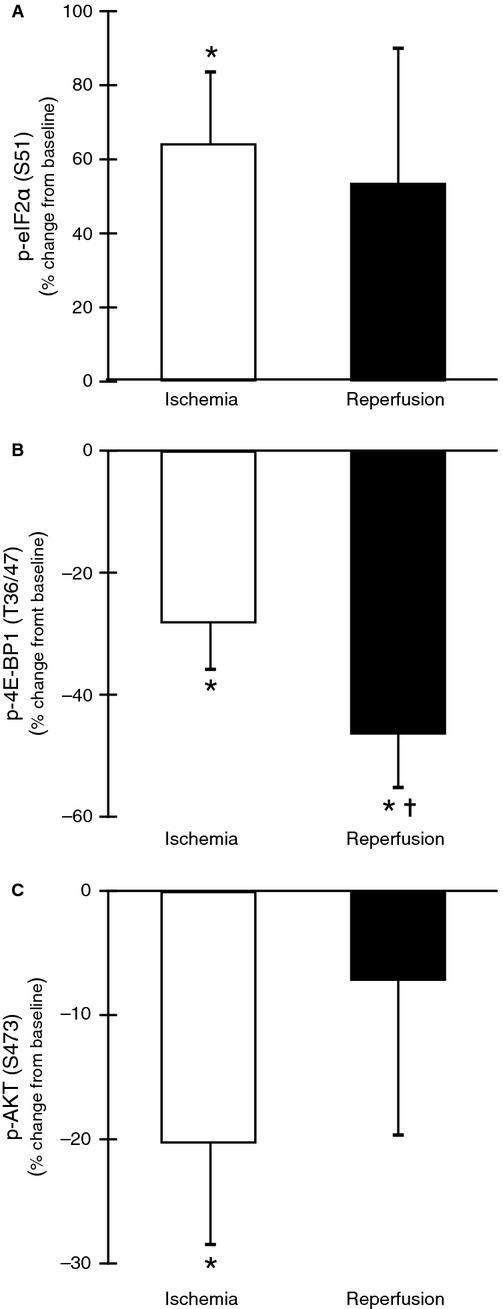
Downregulation of translation during total knee arthroplasty. (A) eIF2α Ser51 phosphorylation increased during ischemia 64% (*P* < 0.05) and showed a trend to increase after reperfusion 53% (*P* = 0.18). (B) Phosphorylated 4E-BP1 at Thr37/46 decreased −28% during ischemia (*P* < 0.05) and was decreased further after reperfusion −46% (*P* < 0.05) compared to baseline (Fig. 1B). Reperfusion decreased 4E-BP1 phosphorylation further −18% compared with ischemia (*P* < 0.05). (C) AKT phosphorylation at Ser473 was decreased −20% in ischemia (*P* < 0.05) and nearly returned to baseline values during reperfusion −7% (*P* > 0.05). Ischemia was not significantly different from reperfusion (*P* > 0.05). *P < 0.05 versus baseline, ^✝^*P* < 0.05 versus ischemia.

Representative blots for each protein of interest are shown in Figure 1. Adequate tissue sample was available to analyze total AKT and 4E-BP1 protein for seven subjects. Total protein levels for AKT did not change at ischemia (*P* > 0.05, 95% CI [−5.7, 60.0]) or reperfusion (*P* > 0.05, 95% CI [−8.70, 14.4]) relative to baseline. Total protein levels for 4E-BP1 did not change at ischemia (*P* > 0.05, 95% CI [−34.4, 152.0]) or reperfusion (*P* > 0.05, 95% CI [−71.6, 65.2]) relative to baseline. Adequate tissue sample was available from all subjects for key components of the ER stress pathway. We observed no significant change in protein levels of BIP during ischemia or into reperfusion. Phosphorylation of eIF2α at Ser51, a potent regulator of translation initiation, increased 64% in ischemia (*P* < 0.05, 95% CI [19.15, 108.90]), however, the effect decreased during reperfusion showing a nonsignificant 53% increase (95% CI [−29.28, 136.01]) (Fig. 2A). Another regulator of translation initiation, phosphorylated 4E-BP1 at Thr37/46, decreased 28% during ischemia (*P* < 0.05, 95% CI [−45.94, −10.42]) and 46% after reperfusion (*P* < 0.05, 95% CI [−66.69, −26.12]) compared with baseline (Fig. 2B). Compared to ischemia, 4E-BP1 phosphorylation was further decreased 18% after reperfusion (*P* < 0.05, 95% CI [−30.21, −6.24]). Further evidence for decreased translation initiation came from phosphorylation of AKT at Ser473 which decreased 20% in ischemia (*P* < .05, 95% CI [−39.29, −2.21]) and nearly returned to baseline values during reperfusion. However, AKT dephosphorylation during ischemia was not significantly different from reperfusion (Fig. 2C).

**Figure 3:**
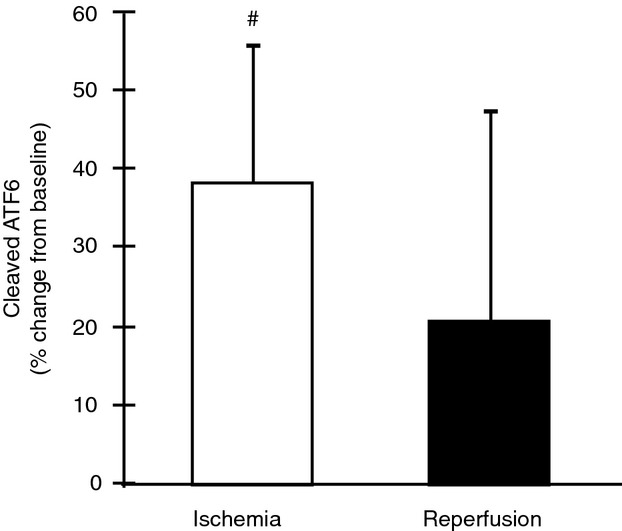
Activating transcription factor 6 cleavage during total knee arthroplasty. Upon release from the endoplasmic reticulum membrane during stress, ATF6 is cleaved into an active 5o-kD fragment. Cleaved ATF6 tended to increase 39% from baseline during ischemia *(P* = 0.056) but was not different from reperfusion (*P >* 0.05). Cleavage of ATF6 during reperfusion was not different from baseline (*P >* 0.05). ^#^*P* < 0.10 versus baseline.

The 50 kD cleaved ATF6 fragment increased 39% during ischemia (*P* = 0.056, 95% CI [−1.08, 78.32]) but was not significantly different during reperfusion (Fig. 3). Downstream, BCL2 showed minimal increases that were nonsignificant during ischemia and during reperfusion (data not shown).

**Figure 4:**
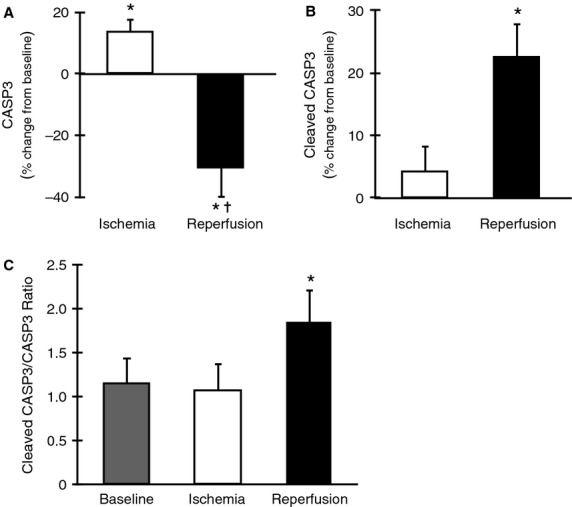
Caspase-3 activation during total knee arthroplasty. (A) Compared to baseline levels full-length CASP3 increased modestly 13% in ischemia (*P* < 0.05) and decreased −30% during reperfusion (*P* < 0.05). From ischemia to reperfusion CASP3 decreased −44% (*P* < 0.05). (B) Levels of cleaved CASP3 (15–17kD) were not different from baseline in ischemia (*P* > 0.05) but increased 23% during reperfusion (*P* < 0.05) and tended to increase compared with ischemia by 18% (*P* = 0.057). (C) The ratio of cleaved to full-length CASP3 was unchanged during ischemia but increased 61.0% during reperfusion (*P* < 0.05). *P < 0.05 versus baseline.

Caspase-7 protein did not change significantly during ischemia or reperfusion. Cleaved CASP7 was not detectable. However, full-length CASP3 increased 13% during ischemia (*P* < 0.05, 95% CI [3.52, 23.75]) and then decreased 30% during reperfusion (*P* < 0.05, 95% CI [−53.12, −7.78]) compared to baseline (Fig. 4A). Compared to ischemia, full-length CASP3 was reduced 44% (*P* < 0.05, 95% CI [−64.24, −23.93]). Cleaved CASP3 was not significantly altered in ischemia but was increased 23% after reperfusion (*P* < 0.05, 95% CI [9.80, 35.36]) compared to baseline (Fig. 4B). The ratio of cleaved to full-length CASP3 increased 61.0% from baseline to reperfusion (*P* < 0.05) but did not change significantly during ischemia (Fig. 4C).

**Figure 5:**
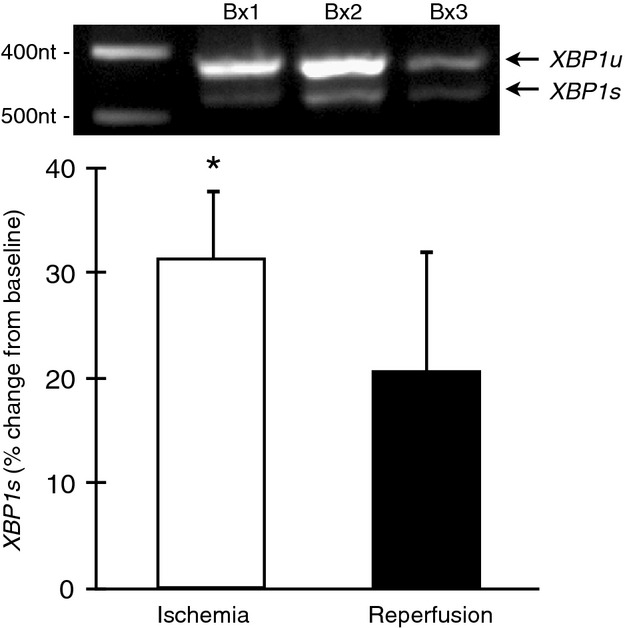
*XBP1* splicing during ischemia. *XBP1* mRNA contains a 26 nucleotide (nt) hairpin that is selectively spliced out by activated IRE1α. The mRNA is reduced from 472 to 448nt. Isolated RNA was amplified by RT-PCR with primers designed around the spliced region. Representative agarose gel showing spliced and unspliced *XBP1*. Data are expressed as mean percent change ± SE (*n* = 8). *P < 0.05 versus baseline.

Total XBP1 protein was unchanged in ischemia and showed a trend to decrease 24% after reperfusion (*P* = 0.094, 95% CI [−52.58, 9.84]) compared to baseline. *XBP1s* RNA content increased 31% during ischemia (*P* < 0.05, 95% CI [−48.90, −13.82]) and was not significantly different after reperfusion (*P* = 0.122, 95% CI [−48.38, 7.08]) while there was no change in *XBP1u* expression in either ischemia or reperfusion (Fig. 5).

## Discussion

During TKA muscle cells are subjected to metabolic perturbations resulting from I/R. We have recently reported on alterations in cell signaling pathways indicating a decrease in cap-dependent translation, initiation, and elongation (Ratchford et al. 2012) and an upregulation of stress-activated protein kinases (JNK) and catabolic activation involving MuRF1 and MAFbx (Bailey et al. 2012). Our present objective was to further characterize the effects of I/R by measuring proteins involved in the UPR and ER stress response in muscle cells during and immediately after surgery. We hypothesized that ischemia and reperfusion during TKA would activate the UPR in response to ER stress.

Our data suggest that all three branches of the ER stress response are upregulated during ischemia: cleaved ATF6 increased, phosphorylation of eIF2α and dephosphorylation of 4E-BP1 indicate PERK activation, and XBP1 splicing suggests induction of IRE1α. These three branches of the UPR initially act to moderate protein synthesis and selectively increase the protein folding capacity of the ER through transcriptional activation. Furthermore, more caspase 3 under the cleaved form and a decreased phosphorylation of 4E-BP1 through reperfusion may indicate a prolonged stress response after TKA.

Confirming our previous results, regulators of translation initiation were downregulated during ischemia and reperfusion (Ratchford et al. 2012). Under conditions of ER stress PERK is released from the ER membrane and activated. PERK phosphorylates eIF2α at Ser51 decreasing the efficiency of translation initiation (Wek and Cavener 2007). During ischemia we found a 64% increase in p-eIF2α that may continue through reperfusion. eIF2α selectively enhances ATF4 translation which in turn can induce 4E-BP1 expression. We have previously measured an increase in ATF4 protein content during ischemia (Ratchford et al. 2012) and hypophosphorylation of 4E-BP1 (Bailey et al. 2012; Ratchford et al. 2012). p-4E-BP1 has been shown to be hypophosphorylated during ER stress in muscle (Deldicque et al. 2011). We measured a 28% and 46% reduction of p-4E-BP1 (Thr37/46) during ischemia and reperfusion, respectively, indicating further dephosphorylation (inhibition of translation initiation) with reperfusion. ER stress has also been linked to decreases in protein synthesis through inactivation of mTORC1 (Deldicque et al. 2010) and 4E-BP1 dephosphorylation (Teleman et al. 2005). Our data show a 20% decrease in pAKT (Ser473) during ischemia but a return to baseline levels after reperfusion is consistent with our previous findings (Bailey et al. 2012). Increased AKT phosphorylation induces activity of mTORC1 through direct phosphorylation or modulation of positive and negative effectors of mTORC1 (Inoki et al. 2002; Vander Haar et al. 2007; Avery et al. 2010; Nascimento et al. 2010). During ER stress a decrease in AKT signaling may in turn decrease mTORC1 activity to reduce global protein synthesis as shown by Deldicque et al. (2010, 2011) in mouse skeletal muscle and C2C12 cells. Furthermore, AKT is thought to mediate ER stress-induced apoptotic pathways as well as ER stress-related anabolic resistance (Yung et al. 2007; Deldicque et al. 2011).

IRE1α activation is proposed by the existence of an increase in *XBP1s* during ischemia and reperfusion. *XBP1* mRNA contains a small hairpin structure that is spliced out of the mature RNA and results in a functional transcription factor protein involved in the UPR and is a strong marker of induction of the UPR. *XBP1s* increased 31% during ischemia and showed a trend to remain increased (20%) through reperfusion. The uncleaved transcript, *XBP1u*, tended to increase but was not significantly different from baseline levels. The RNase activity of IRE1α is also linked to translation attenuation through targeted *28s* rRNA cleavage (Iwawaki et al. 2001). ER stress activation of IRE1α is also linked to JNK activation (Urano et al. 2000) which was shown in skeletal muscle during TKA-induced ischemia (Bailey et al. 2012). Consistent with these reports, JNK activation was also shown in mouse skeletal muscle in response to ER stress activated by a high fat diet (Deldicque et al. 2010). IRE1α also plays a role in the activation of ER membrane associated caspases and the progression toward apoptotic pathways if ER stress is not alleviated. CASP3 has been shown to play an important role in initializing muscle protein degradation in catabolic conditions (Du et al. 2004). During ischemia procaspase-3, was increased 13% but decreased 30% from baseline during reperfusion. Concomitantly, cleaved CASP3 increased 23% during reperfusion suggesting activation of CASP3 upon reperfusion.

The third branch of the UPR, ATF6, was activated as shown by a 39% increase in cleaved ATF6 at maximal ischemia but not during reperfusion. The activation of ATF6 in ischemia and inactivation after reperfusion has been demonstrated previously (Doroudgar et al. 2009) and may be a mediator of I/R preconditioning. After cleavage the newly activated transcription factor induces UPR genes including *XBP1*, *BIP*, and *CHOP*. CHOP produces a transcription factor involved in regulating the BCL-2 family of apoptosis regulating proteins. Contrary to a previous report (Bailey et al. 2012), BCL2 levels did not change during ischemia or reperfusion. However, our present analysis utilized whole-muscle homogenates versus cytoplasmic and nuclear fractions (Bailey et al. 2012).

ER stress is activated by impaired ER protein folding due an accumulation of proteins or inability to fold proteins caused by dysregulation of glycosylation, disulfide bond formation, or calcium levels all of which have been observed to change in response to hypoxia or ischemia (Allen and Orchard 1983; Tanaka et al. 2000; Shimizu and Hendershot 2009; Groenendyk and Sreenivasaiah 2010; Shirato et al. 2011). Exsanguination and tourniquet use eliminates oxygen and nutrient delivery and initiates a cascade of events that may potentiate ER stress starting with a shift in metabolism toward glycolysis. In combination with a lack of glucose delivery and byproduct removal, these factors cause local glucose concentrations decrease to less than half their original levels in skeletal muscle after 20 min of ischemia while lactate concentration nearly doubles (Korth et al. 2000). Also, due to anesthesia, patients are in a fasting state. The change in energy status of the muscle cell may acutely alter calcium regulation and glycosylation both of which are necessary for adequate protein folding in the ER. Furthermore, protein-disulfide isomerase requires oxygen therefore during ischemia proper protein folding may be inhibited (Gorlach et al. 2006).

Limitations to the study include the lack of direct measures of oxygen tension, calcium levels, and energy status during ischemia and reperfusion. Additionally, the inclusion of a nontourniquet control group would help us to better interpret our results in light of the fact that the biopsy procedure itself may be influencing these pathways. Furthermore, I/R can result in oxidative stress, inflammation, and mitochondrial disfunction that may stimulate ER stress and UPR signaling. However, we are unable to comment on the specific contributions of different pathologic phenomena toward the progression of ER stress during TKA and suggest further studies to investigate the duration and functional consequences of cell signaling changes during and after TKA. Lastly, the amount of tissue obtained during each muscle biopsy limits the scope of our analysis.

In conclusion, we have shown that during and immediately after TKA there is a decrease in anabolic signaling (Ratchford et al. 2012), an upregulation of catabolic pathways (Bailey et al. 2012), and bearing in mind the aforementioned limitations, now include the present findings suggesting the induction of ER stress. While further work is needed in order to measure changes at later time points as well as following anabolic stimulus, essential amino acid ingestion (Dreyer et al. 2008), we interpret the current findings as potentially contributing to the proximate signals that may initiate a cascade of events that lead to muscle loss measured within 2 weeks of surgery.

### Perspectives & significance

Within the next two decades, the total number of TKAs performed annually in the U.S., is projected to increase to 3.5 million (Kurtz et al. 2011). While TKA has proven to be a effective surgical remediation for chronic knee pain associated with OA, several studies have suggested that there is a long-term inability to regain muscle mass in older adults following TKA (Finch et al. 1998; Walsh et al. 1998; Mizner and Snyder-Mackler 2005; Mizner et al. 2005a,b; Yoshida et al. 2008) and that atrophy is the greatest contributor to functional mobility impairments (Meier et al. 2008, 2009). In the first 2 weeks after surgery TKA patients lose approximately 12% of quadriceps muscle volume (Ratchford et al. 2012). As such, insight into proximate causes of atrophy following TKA are warranted in view of the fact that current outcomes exchange chronic knee pain for muscle loss, which in older TKA patients may be permanent. While there is no question that eliminating knee pain is the most appropriate course of action further research is needed in order to promote muscle recovery or, better yet, prevent loss during this critical time so that chronic mobility impairments are reduced.
